# Simultaneous elimination of Malachite Green, Rhodamine B and Cresol Red from aqueous sample with Sistan sand, optimized by Taguchi L16 and Plackett–Burman experiment design methods

**DOI:** 10.1186/s13065-018-0486-2

**Published:** 2018-11-16

**Authors:** Sahar Marghzari, Mojtaba Sasani, Massoud Kaykhaii, Mona Sargazi, Mohammad Hashemi

**Affiliations:** 1 Department of Chemistry, Faculty of Sciences, University of Sistan and Baluchestan, Zahedan, 98155-674 Iran; 2 Young Researchers and Elite Club, Zahedan Branch, Islamic Azad University, Zahedan, Iran; 3 Smartphone Analytical Sensors Research Centre, University of Sistan and Baluchestan, Zahedan, Iran; 4 Department of Clinical Biochemistry, School of Medicine, Zahedan University of Medical Science, Zahedan, Iran

**Keywords:** Simultaneous removal of dyes, Taguchi design, Plackett–Burman design, Malachite green, Rhodamine B, Cresol red, Sand

## Abstract

The purpose of this study was to investigate the feasibility of simultaneous optimization and removal of dyes, Malachite green (MG), Rhodamine B (RhB) and Cresol Red (CR) from aqueous solutions by using Sistan sand as an extremely low cost adsorbent. Factors affecting adsorption of the analytes on the sorbent were investigated experimentally and by using Taguchi and Plackett–Burman experimental design methods. In most cases, the results of these two models were in agreement with each other and with experimental data obtained. Taguchi method was capable to predict results with accuracies better than 97.89%, 95.43%, and 97.79% for MG, RhB, and CR, respectively. Under the optimum conditions, the sorbent could remove simultaneously more than 83% of the dyes with the amount of adsorbed dyes of 0.132, 0.109, and 0.120 mg g^−1^ for MG, RhB and CR on sand, respectively. Kinetic studies showed that pseudo second order is the best model of adsorption for all analytes. Thermodynamic parameters revealed that this process is spontaneous and endothermic.

**Electronic supplementary material:**

The online version of this article (10.1186/s13065-018-0486-2) contains supplementary material, which is available to authorized users.

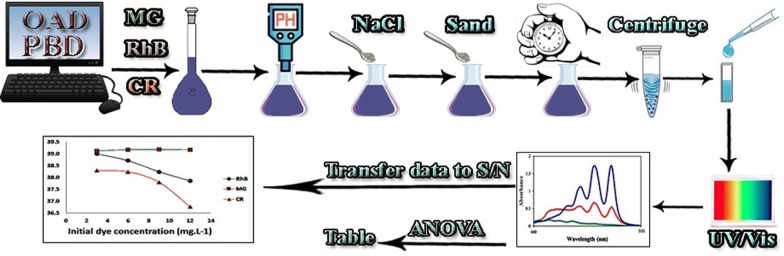

## Introduction

Industrial wastewater is one of the major pollutants of the environment. Colored wastewaters are produced in many industries such as textile, pharmaceutical, food, cosmetic and leather industries [1, 2]. Annually, more than 10,000 metric tons of dyes are consumed in textile industries which makes their wastewater as one of the most important environmental pollutants [3]. Typically, the main pollutant in textile wastewater is organic dyes which many of them are resistant to biodegradation. Moreover, colored wastewater prevents the penetration of sunlight into the water and reduces the speed of photosynthetic process [4–7]. More importantly, their carcinogenic effects and genetic mutations in living organisms are proved [8, 9]. Therefore, it is of importance to maintain human and environmental healthy by removing dyes using cheap and economical methods. Various methods have been evaluated for this purpose, such as electrochemical coagulation, using membranes, photocatalytic techniques, electrochemical methods, biological processes and adsorption techniques [3]. Since adsorption process is the most economical method and has a simpler operational capability, in most cases, it is preferred to other techniques [10, 11]. Nano-particles are of high interest for simultaneous removal of dyes nowadays. For example, cobalt hydroxide nano-particles were applied for simultaneous removal of Indigo Carmine and Methyl orange [12]. In another study, four toxic dyes including Brilliant Green, Auramine O, Methylene Blue and Eosin Yellow were removed by CuO Nano-particles loaded on activated carbon [13]. While nano-particles show good performance and high capacity, synthesis of them needs high skill and pure materials are needed; so, most of these materials are not produced in large quantities. Consequently, they are not available in sufficient bulk to be commercialized for full-scale application. Because of these drawbacks, many researchers tried to find cost-effective adsorbents to eliminate dyes [14, 15]. Natural sands contain active components that can strongly adsorb positively charged organic material from an aqueous solution. The potential of using sand for this purpose has been studied and results were promising [16, 17]. However, we could not find any report on applying sand for simultaneous removal of dyes.

For optimization of the parameters affecting adsorption efficiency, it is very common to use one-factor-at-a-time (OFAT) method, in which all parameters are keeping constant while one factor is optimized. In this method, it is assumed that each parameter is completely independent of the others. There are obvious advantages for design of experiment (DOE) methods over OFAT, including less resource requirements; ability to assess the effect of factors precisely; and finally by this method, interaction between factors is not neglected [18–20]. Taguchi method is one of these DOE methods which is mainly developed for optimization. By using Taguchi method, the impact of each controllable factor can be determined as well [21]. Plackett–Burman Design (PBD) is a well-established and widely used statistical technique for selecting the most effective components affecting adsorption process with high significance levels for further optimization [22].

In this study, very cheap sand sorbent is used for simultaneous removal of three dyes, Malachite green, Rhodamine B and Cresol Red from water samples and in order to find the optimum conditions for this process, Taguchi design was used. This method selected because it has some advantages over other traditional uni-variant optimization techniques, including less number of experiments is required [23–25]. Moreover, Plackett–Burman design was also applied for the same purpose and results were compared to Taguchi design. ANOVA was used to determine and confirm the results obtained experimentally.

## Experimental

### Instruments and materials

Sand which was used in this study as dye sorbent was collected from Sistan desert, south east of Iran. MG (catalog number 1013980025), RhB (catalog number 1075990025) and CR (catalog number 1052250005) dyes were purchased from Merck KGaA, Darmstadt, Germany. Table 1 shows physical and chemical characteristics of these adsorbates. Other solvents and reagents were purchased from Fluka AG (Switzerland). Stock solutions of dyes were prepared by dissolving 0.5 g of each dye in distilled water in 1000 mL volumetric flasks. The test solution containing a mixture of MG, RhB and CR were prepared daily by diluting the proper volume of stock solution in deionized water. pH meter (model EasySeven, Metrohm, Switzerland) was applied to measure the pH of sample solutions. In order to determine the residual concentration of dyes after adsorption, UV–Vis spectrophotometer (model Lambda 25, Perkin Elmer Corp., USA) was used. Sistan sands were characterized by scanning electron microscope (SEM, model EM3900M, KYKY, China) and Fourier transform (FT-IR) spectroscopy (Spectrum two FTIR, Perkin Elmer Corp., USA). Minitab 16 and Qualitek 4 softwares (version 14.7.0) were used for PBD and Taguchi design methods, respectively.

**Table 1:** Physical and chemical characteristics of adsorbates

Dye	Chemical structure	Molecular weight (g mol^−1^)	λ_max_ (nm)
Malachite green		364.92	618
Rhodamine B		479.02	554
Cresol red		382.43	425

### Analytical procedure

**Fig. 1:**
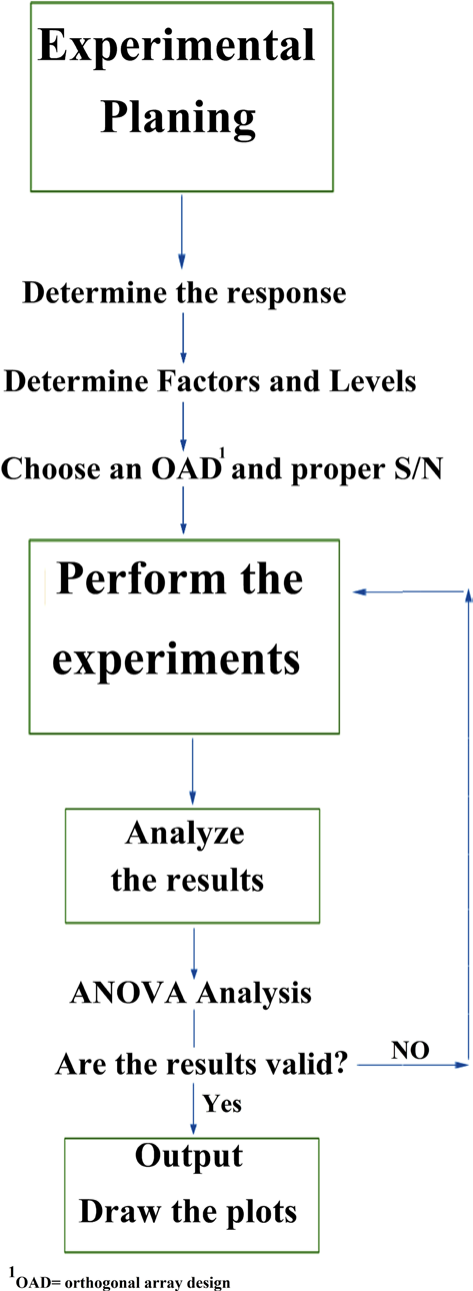
Procedure of Taguchi design method

In order to study the efficiency of simultaneous removal of MG, RhB and CR by sand, batch technique was used for their adsorption; and to optimize parameters affecting adsorption, design experiments according to Taguchi design L16 was employed (Fig. 1). Experiments were performed in 6 steps: (1) 20 mL solution of 3-dyes mixture, with the concentrations mentioned in Additional file 1: Table S1, was prepared in a 50 mL flask. (2) pH of the sample solution was adjusted either by 0.1 M HCl or 0.1 M NaOH. (3) Appropriate amounts of NaCl and adsorbent were added to the flask carefully. (4) Sample was shaked on a shaker for a preset time to reach equilibrium state. (5) This mixture was centrifuged for 10 min at 5000 rpm (1957 relative centrifugal force) to separate adsorbent particles from the solution and supernatant liquid were collected. (6) The concentration of dyes remained in the sample after removal of the dyes, was determined spectrophotometerically against a blank in the wavelengths mentioned in Table 1. External calibration curves were used.

After then, the percentage of each dye adsorbed was calculated using equation (1) 12]:

1 $${\text{\% Removal = }}\frac{{C_{0} - C_{e} }}{{C_{0} }} \times 100$$

where C_e_ and C_0_ are equilibrium and initial dyes concentration (mg L^−1^) respectively.

In adsorption studies, q_e_ (mg g^−1^) is the amount of adsorbed dye on sorbent in equilibrium state and it can be calculated according to equation (2) [26]:

2 $$q_{e} = \frac{{(C_{0} - C_{e} ) \times V}}{m}$$

where C_0_ and C_e_ (mg L^−1^) are respectively the concentration of dyes at initial point and at equilibrium, V (L) is the volume of the solution and m (g) is the mass of dry adsorbent used.

### Taguchi design of experiments

Figure 1 depicts the experiments design procedure [27, 28]. Analysis of variances (ANOVA) and signal to noise (S/N) ratio (SNR) are two main statistical methods which can confirm the results obtained by Taguchi method [29]. SNR is a ratio of mean response (as signal) to standard deviation (as noise) [30]. In this way, bigger S/N is desirable and bigger characteristic for S/N formula is defined as equation (3) [31]:

3 $$\frac{{\text{S}}}{{\text{N}}} = \frac{{ - 10 {\text{Log }}\left(\frac{1}{{{\text{y}}_{1}^{2} }} + \frac{1}{{{\text{y}}_{2}^{2} }} + \cdots + \frac{1}{{{\text{y}}_{\text{n}}^{2} }}\right)}}{\text{n}}$$

where n is number of replications s, and y_i_ is the response of detector.

Since the process of simultaneous removal of MG, RhB and CR was desired, 5 factors in 4 levels were chosen and L16 was offered by Qualitek 4 (Table 2). Consequently, 16 experiments were designed. Additional file 1: Table S1 shows the factors and levels which were used in these set of experiments. After doing experiments, optimum levels for each factor were determined by S/N and mean of mean (Table 3).

**Table 2:** Taguchi design and obtained results for simultaneous removal percentage of MG, RhB and CR

No	pH	Adsorbent dosage	NaCl added	Contact time	Initial dye concentration	MG1 (%)	MG2 (%)	MG3 (%)	CR1 (%)	CR2 (%)	CR3 (%)	RhB1 (%)	RhB2 (%)	RhB3 (%)
1	1	1	1	1	1	81	85	79	64	64	67	88	87	83
2	1	2	2	2	2	88	82	81	80	79	74	89	87	83
3	1	3	3	3	3	86	86	85	79	83	73	86	88	83
4	1	4	4	4	4	83	83	78	68	66	71	79	75	80
5	2	1	2	3	4	82	87	81	56	56	58	82	76	75
6	2	2	1	4	3	93	92	91	71	66	67	90	87	90
7	2	3	3	1	2	92	86	97	94	86	87	77	76	80
8	2	4	3	2	1	93	94	93	95	88	99	87	81	83
9	3	1	3	4	2	87	80	91	73	75	68	86	81	89
10	3	2	4	3	1	93	89	93	91	93	87	87	83	86
11	3	3	1	2	4	97	89	99	70	72	67	82	75	79
12	3	4	2	1	3	97	98	93	82	77	84	79	73	75
13	4	1	4	2	3	87	80	83	76	78	74	73	75	72
14	4	2	3	1	4	96	92	97	82	80	75	71	74	72
15	4	3	2	4	1	94	98	98	80	79	80	94	92	97
16	4	4	1	3	2	96	90	92	79	73	77	94	86	86

**Table 3:** Optimum conditions for each factor to simultaneous removal of MG, RhB and CR

	pH	Adsorbent dose	NaCl added	Contact time	Initial dye concentration
S/N	3	2	3	2	3
Mean of mean	3	2	3	2	3

## Results and discussion

### Morphology and characterization of adsorbent

**Fig. 2:**
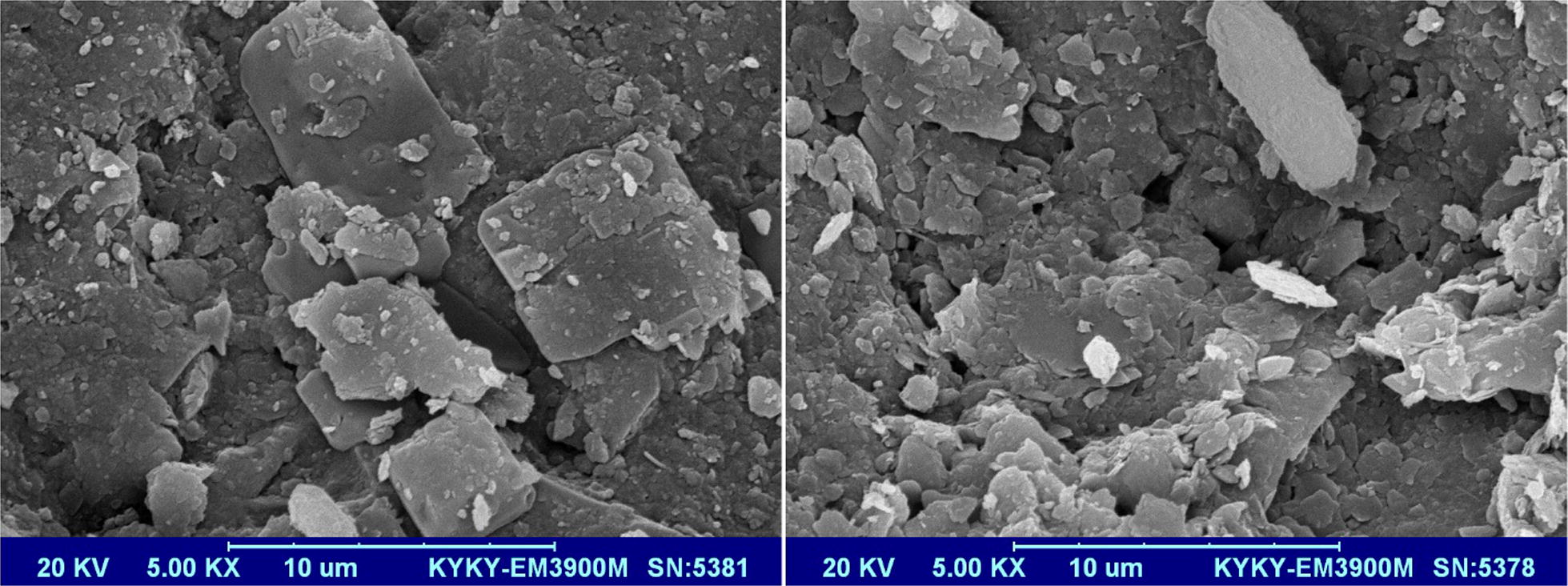
SEM image of Sistan sand

As can be seen in scanning electron microscope (SEM) image of Sistan sand (Fig. 2), it has an irregular and fractured surface structure. The average size of adsorbent particles was 250 µm which was determined using Image^J^ software. The FT-IR spectrum of sand (Additional file 1: Figure S1) shows a main peak at 1004 cm^−1^ which refers to quartz. Presence of quartz is also proved by absorption bands at 1004, 776, 695, 531 and 462 cm^−1^. A peak at 2347 cm^−1^ can be assigned to silane [32].

### Effect of factors affecting concurrent adsorption of MG, RhB and CR

To obtain the best performance of the adsorption process for simultaneous removal of three target dyes and achieving satisfactory efficiency in the shortest possible time, several parameters influencing adsorption were studied and optimized while all target compounds were exist in the sample solution. The parameters studied were the amount of sorbent, pH of sample solution, effect of contact time, ionic strength of the sample solution, and initial concentration of each dye. Each experiment was run in triplicates.

#### Effect of pH

**Fig. 3:**
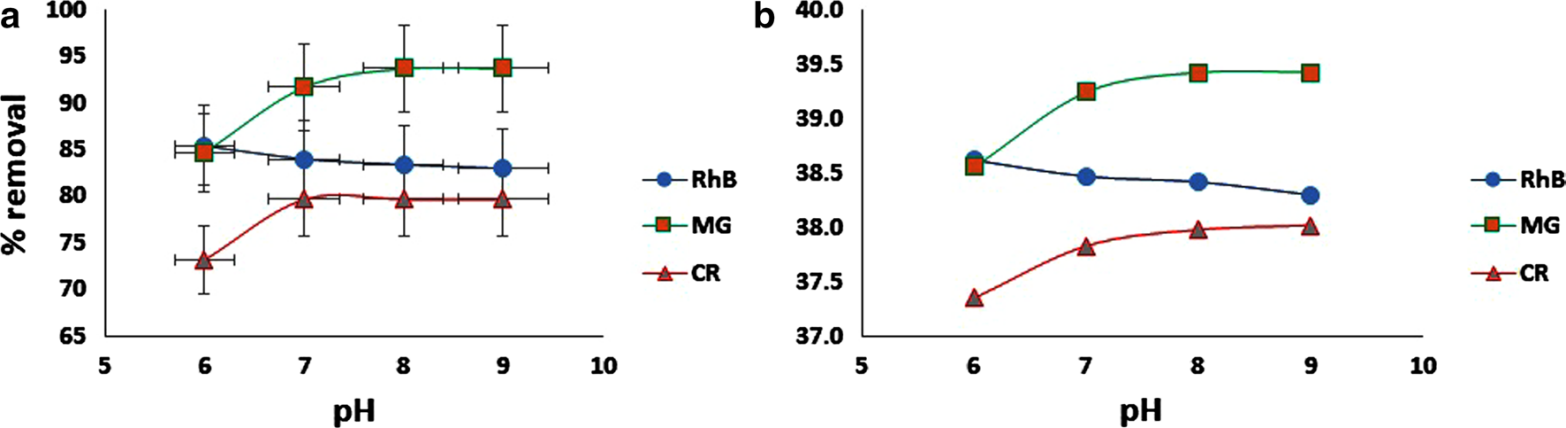
Effect of pH on removal of MG, RhB and CR based on Mean (**a**) and S/N (**b**)

Initial pH of sample solution has a great effect on adsorption capacity. In order to find the effect of pH on simultaneous adsorption of MG, RhB and CR on Sistan sand, pH of solutions were varied between 6 and 9. Figure 3 represents the results of simultaneous dye removal based on mean and S/N versus pH. As can be seen, optimum pH is 8.0 in level 3. For CR and MG, the optimum pH is falling at basic pHs due to the formation of negative charges on the adsorbent surface; and at the same time, protonation of these two dyes [33]. For RhB, the adsorption is high in acidic media and decreases with the increase in pH of the solution. It can be interpreted according to the pK_a_ of RhB which is 3.7. Above this pH, deprotonation of the carboxyl functional group occurs and therefore, an attraction between the carboxylate ion and the xanthene groups of the RhB results in the formation of dimers of the dye which results decreasing in adsorption, however this decrement is not very sharp in the pH interval we studied [34].

#### Effect of adsorbent dosage

**Fig. 4:**
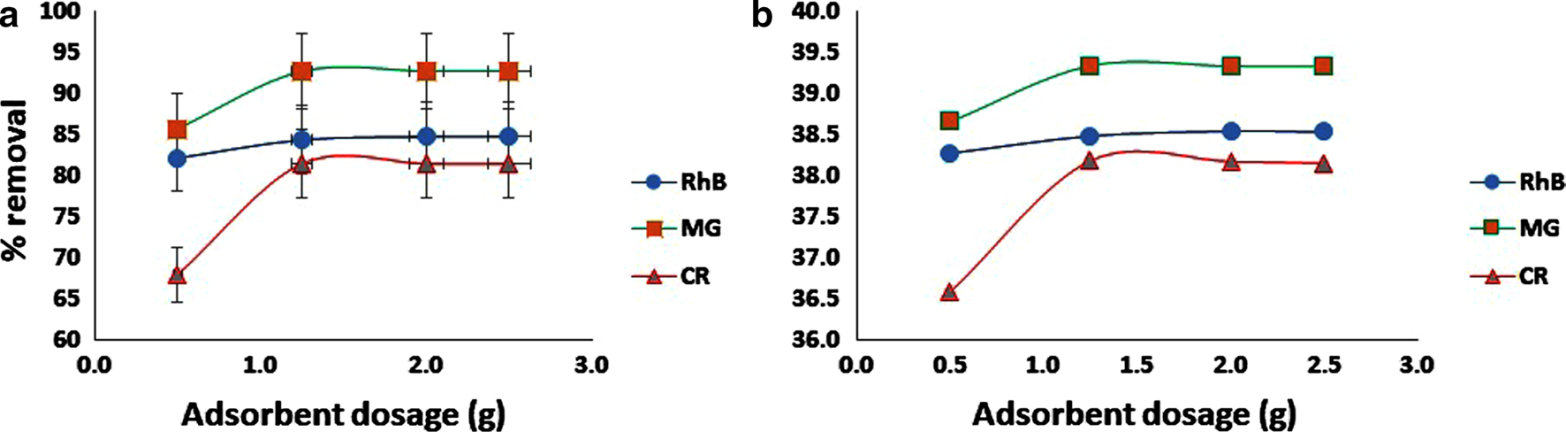
Effect of adsorbent dosage on removal of MG, RhB and CR based on Mean (**a**) and S/N (**b**)

What is illustrated in Fig. 4 is the effect of adsorbent dosage on percent of simultaneous removal of MG, RhB and CR dyes. As can be seen, due to the increment of the available sorption sites, percent of dye removing increases with increasing of adsorbent dosage. In order to study this effect by Taguchi method, experiments were designed with 4 levels of adsorbent in the range of 0.5–2.5 g. The optimum level for this factor is second level [23].

#### Effect of ionic strength

The salting-out effect is widely applied in traditional liquid–liquid extraction because it makes the solubility of organic targets in the aqueous phase decrease; thus, more analytes enter into extracting phase. In this study, the influence of salt on the adsorption process was studied at the presence of sodium chloride within the concentration range of 0.025 to 0.100 g mL^−1^. It was observed that changing the ionic strength has different effect on adsorption of different dyes (Additional file 1: Figure S2). By increasing the amount of NaCl, the efficiency of removal of CR increased, while for the two other dyes, the efficiency was decreased. Due to the competition between cationic dyes (MG, RhB) and Na^+^ ions toward the available adsorption sites, by increasing the ionic strength, the activity of the dyes and the active sites of the sand decreases; hence, the amount of adsorption decreases [35]. On the other hand, for CR, any increase in the ionic strength of the solution leads to the repulsive electrostatic attraction, which leads to adsorption increase [36]. Optimum level for this factor was selected in level 3.

#### Effect of contact time

Removal of dyes by sand was carried out after 10, 20, 30 and 40 min of starting the adsorption process. Results are shown in Additional file 1: Figure S3. For RhB, when contact time increases, removal percent goes up and finally reaches to a constant level which deals with reaching equilibrium after 30 min. However, for the two other dyes, after passing 20 min, the adsorption decreases. To have a balance for all dyes, the optimized contact time was selected at 20 min or second level. This phenomena occurs, probably due to the fact that while an equilibrium is attained, RhB can win the competition for available sites on the sand in long term.

#### Effect of initial dye concentration

Additional file 1: Figure S4, which is shown in supplementary data, shows the effect of initial dye concentration on simultaneous adsorption of the analytes on sand. To evaluate the effect of initial dye concentration solution were made which contain concentrations between 3 and 12 mg L^−1^ of each dye. It was found that by increasing the initial dye concentration, the efficiency reduces because of limited active site available on the sorbent [37]. The optimum conditions for this parameter selected 9 mg L^−1^ in level 3.

### Optimization process

Participation and importance of each optimized factor was determined by ANOVA. In all factors, the optimal levels obtained through S/N and the means are normally equal. An ideal result is one with the highest S/N ratio [38]. Table 3 shows optimum levels for each factor. In order to verify that Taguchi has a perfect ability for response prediction., a comparison between predicted and practical results was performed. Results are mentioned in Table 4. In order to check the performance of prediction of Taguchi design method in this process, compliance percent is calculated according to equation (4):

4 $${\text{Compliance percent}} = \frac{\text{Practical result}}{\text{Predicted values}} \times 100$$

Pure sum of square for a particular factor is calculated according to the following equation (5) [23]:

5 $${\text{pure sum}} = {\text{sum of square}} = V_{A} \times DOF$$

where V_A_ is the variance of A. ANOVA Analysis of variance was used to evaluate the orthogonal array of design results and is presented in Additional file 1: Table S2. The last column in the Table shows the contribution of each factor to the adsorption process.

**Table 4:** Practical and predicted values for dyes removal by using Taguchi method

Dyes	Predicted (%)	Practical (%)	Compliance percentage (%)
MG	93.98	96	97.89
RhB	78.25	82	95.43
CR	88.01	90	97.79

### Plackett–Burman design

In order to screen and find the best conditions for simultaneous removal of dyes, a Plackett–Burman design which is a multivariate strategy, was used. PBD is a two-level partial factorial design that can be used as an excellent screening tool to extract important information about the main factors affecting the system under study [39, 40]. Here, it was used to identify the most effective parameters involved in the simultaneous adsorption of dyes. For this purpose, 5 factors were investigated in 2 levels. Additional file 1: Table S3 shows the factors and levels at low (− 1) and high (+ 1) levels of PBD. This method was designed by Minitab 16 software.

**Fig. 5:**
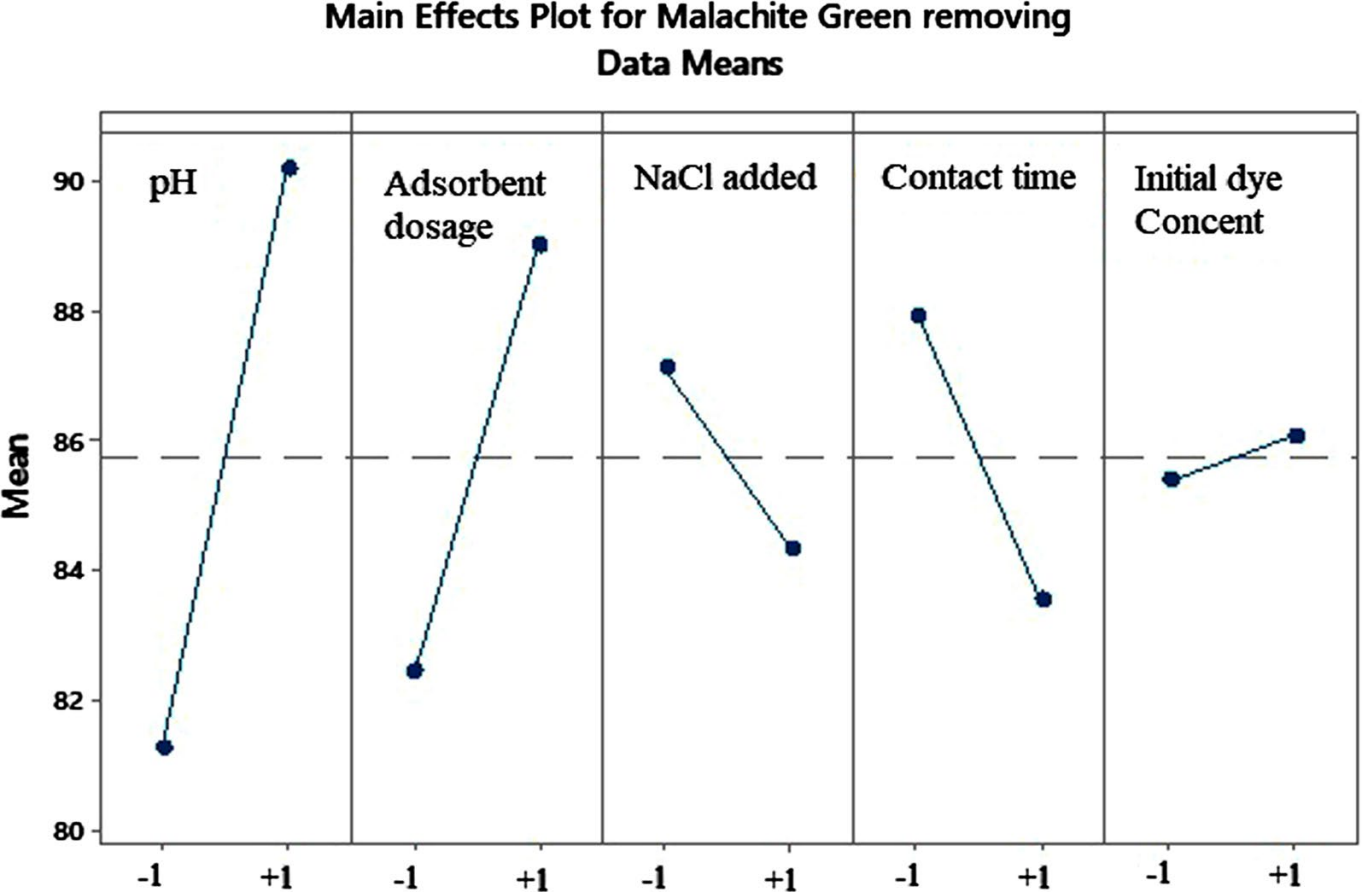
Main effects plot for MG removal by PBD

Results of experimental design for 12 experiments in 5 factors are plotted in Fig. 5, Additional file 1: Figures S5 and S6. Table 5 compares the priority of each of the factors studied in the PBD and Taguchi designs and reflects the conformance of the two methods.

**Table 5:** The effectiveness of factors in PBD and Taguchi design

Effectiveness	Plackett-Burman design			Taguchi design		
	MG	RhB	CR	MG	RhB	CR
1	pH	Initial dye concentration	Initial dye concentration	Adsorbent dosage	Initial dye concentration	Adsorbent dosage
2	Adsorbent dosage	Ionic strength	Adsorbent dosage	pH	Contact time	Initial dye concentration
3	Contact time	Contact time	Ionic strength	Ionic strength	Ionic strength	Ionic strength
4	Ionic strength	Adsorbent dosage	Contact time	Initial dye concentration	Adsorbent dosage	Contact time
5	Initial dye concentration	pH	pH	Contact time	pH	pH

### Kinetic study of adsorption

In order to find the mechanism of adsorption of dyes on the sand, different kinetic models have been examined. The adsorption rate can be also predicted from kinetic parameters [41]. Eight experiments were carried out by OFAT method to study kinetic models. In this set of experiments, contact time was changed in the range of 1–30 min and other variables including pH, adsorbent dosage, initial dye concentration and amount of NaCl were kept constant at their optimum level. Results of these experiments were investigated with the following pseudo first-order equation (6):

6 $$Log \left( {q_{e} - q_{t} } \right) = Log q_{e} - \left( {\frac{{K_{1} }}{2.303}} \right)t$$

where the amount of dye adsorbed at any time is shown as q_t_ (mg g^−1^), t is contact time (min) and the pseudo-first order constant is K_1_ (min^−1^) [42]. By plotting the log (q_e _− q_t_) versus t, K_1_ and q_e_ were calculated from the slope and intercept of the plot, respectively. Pseudo second order was calculated by equation (7):

7 $$\frac{t}{{q_{t} }} = \frac{1}{{K_{2} q_{e}^{2} }} + \frac{1}{{q_{e} }}t$$

The adsorption rate constant of this model, K_2_ (g mg^−1^ min^−1^) is the pseudo-second order constant which was obtained from the intercept of the plot of t/q_t_ against t. The slope of this plot shows q_e_ [43]. Additional file 1: Table S4 presents the kinetic parameters for simultaneous adsorption of MG, RhB and CR on Sistan sand, and reveals that pseudo second order is the best fitted model for kinetic of removal of them. A similar observation is reported in adsorption of reactive orange 16 [44].

### Thermodynamics studies

The thermodynamic parameters such as changing the enthalpy (ΔH°), entropy change (ΔS°) and Gibbs free energy (ΔG^°^) represent some information which confirms adsorption nature and are useful to evaluate the feasibility and the spontaneous nature of adsorption. Van’t Hoff plot (Eq. 8) was used to calculate ΔH° and ΔS° of each dye adsorbed on the sand from the slope and intercept of this plot, respectively.

8 $$\log \left( { \frac{{q_{e} }}{{C_{e} }}} \right) = \frac{{\Delta {\text{S}}^\circ }}{2.303 R} - \frac{{\Delta {\text{H}}^\circ }}{2.303RT}$$

where R (8.304 J mol^−1^ K^−1^) is the universal gas constant and T is the absolute temperature of the solution (K).ΔG° was calculated from equation (9) [45]:

9 $$\Delta {\text{G}}^\circ = \Delta {\text{H}}^\circ - {\text{T }}\Delta {\text{S}}^\circ$$

In order to determine the thermodynamic parameters of simultaneous removal of MG, RhB and CR, 4 experiments were carried out by OFAT method. All experimental conditions were kept constant and temperature was varied. What are tabulated in Additional file 1: Table S5 are the values of the above parameters. It is clear that positive ΔH° represents that the adsorption process is endothermic. Positive ΔS° reveals that there is an increase in randomness between the 2 phases (solid/liquid) in solution. According to the values obtained for ΔG°, the spontaneous of the simultaneous adsorption of three dyes by Sistan sand is confirmed. Total values of the thermodynamic parameters reveal that this process take place through electrostatic interactions [46].

### Real sample analysis

In order to study the efficiency of the method for simultaneous removal of MG, RhB, and CR from water samples, a 20 mL aliquot of tap water was spiked with 9 mg L^−1^ of each dye. Sistan sand was applied as adsorbent under optimal conditions. Spectrophotometry showed that the percentage removal of dyes for MG, RhB, and CR obtained were 92%, 76% and 83%, respectively. Also, using equation [2], q_e_ for MG, RhB, and CR was calculated to be 0.133, 0.109, and 0.120 mg of dye per g of the sand, respectively. In Table 6, some other sorbents reported in the literature were compared with the Sistan sand for the adsorption of the same organic dyes. While the most of the other sorbents need pretreatments or modifications, Sistan sand which is costless and is plenty available, still has good performance for simultaneous removal of dyes.

**Table 6:** A comparison on removal of MG, RhB and CR by different adsorbents

No	Adsorbent	Adsorbate	qe (mg g^−1^)	References
1	Sahara desert sand	Methylene Blue	11.98	[47]
2	Feldspar	Methylene Blue	0.66	[48]
3	Bentonite	MG	7.72	[49]
4	3A zeolite	RhB	0.74	[23]
5	Zeolite MCM-22	RhB	1.11	[50]
6	Beach sand coated with polyaniline	Methylene Blue	9.10	[51]
7	Gypsum	Methylene Blue	36.00	[52]
8	functionalized multi walled carbon nanotubes	MG	114.11	[53]
9	Albizzia lebbeck seed activated carbon	CR	5.154	[54]
10	magnetic Fe_3_O_4_/C core–shell nanoparticles	CR	11.22	[55]
11	Sistan sand	Simultaneous removal of MG, RhB and CR	0.36	This study

## Conclusion

In this study, Sistan sand as a costless and accessible sorbent was used for simultaneous removal of three dyes Malachite green, Rhodamine B and Cresol red from water sample. Optimum conditions for adsorption was designed and predicted by Taguchi method and was determined experimentally. Plackett–Burman design was used to confirm the Taguchi design and as a screening method to identify the significance of each factor influencing this process. In almost all cases, a good agreement between these Taguchi and PBD was observed. Kinetic studies showed that pseudo second order is the best fitted model for all three analytes. This process is endothermic, as thermodynamic studies showed. We also demonstrated that simultaneous adsorption of environmental pollutants, especially dyes, are plainly achievable, even when the nature of target compounds are different.

## Additional file

**Additional file 1: Table S1.** Factors and levels in Taguchi design to remove MG, RhB and CR. **Figure S1**. FT-IR of Sistan sand. **Figure S2.** Effect of ionic strength on removal of MG, RhB and CR based on Mean (A) and S/N (B). **Figure S3.** Effect of contact time on concurrent adsorption based on Mean (A) and S/N ratio (B). **Figure S4.** Effect of initial dye concentration on simultaneous adsorption based on Mean (A) and S/N ratio (B). **Table S2.** ANOVA results for simultaneous removal of MG, RhB and CR. **Table S3.** Factors and levels were used for concurrent adsorption of MG, RhB and CR in PBD. **Figure S5.** Main effects plot for RhB removal by PBD. **Figure S6.** Main effects plot for CR removal by PBD. **Table S4.** Kinetic parameters of simultaneous removal of MG, RhB and CR by Sistan sand. **Table S5.** Thermodynamic parameters on simultaneous removal of MG, RhB and CR.

## Abbreviations

MG: Malachite Green
RhB: Rhodamine B
CR: Cresol Re
OFAT: one-factor-at-a-time
DOE: design of experiment
FT-IR: Fourier transform
SEM: scanning electron microscope
ANOVA: analysis of variance
S/N: signal to noise
SNR: signal to noise ratio
PBD: Plackett–Burman design
