# Supplementary material for: Simultaneous elimination of Malachite Green, Rhodamine B and Cresol Red from aqueous sample with Sistan sand, optimized by Taguchi L16 and Plackett–Burman experiment design methods
Source: Chem Cent J. 2018 Nov 16;12:116. doi: 10.1186/s13065-018-0486-2 (PMC6768036; doi:10.1186/s13065-018-0486-2)
Supplement: Supplementary file 1 — Additional file 1: Table S1. Factors and levels in Taguchi design to remove MG, RhB and CR. Figure S1. FT-IR of Sistan sand. Figure S2. Effect of ionic strength on removal of MG, RhB and CR based on Mean (A) and S/N (B). Figure S3. Effect of contact time on concurrent adsorption based on Mean (A) and S/N ratio (B). Figure S4. Effect of initial dye concentration on simultaneous adsorption based on Mean (A) and S/N ratio (B). Table S2. ANOVA results for simultaneous removal of MG, RhB and CR. Table S3. Factors and levels were used for concurrent adsorption of MG, RhB and CR in PBD. Figure S5. Main effects plot for RhB removal by PBD. Figure S6. Main effects plot for CR removal by PBD. Table S4. Kinetic parameters of simultaneous removal of MG, RhB and CR by Sistan sand. Table S5. Thermodynamic parameters on simultaneous removal of MG, RhB and CR. [file 13065_2018_486_MOESM1_ESM.docx]

**Additional file:**

**Simultaneous elimination of malachite green, rhodamine B and cresol red from aqueous sample with Sistan sand, optimized by Taguchi l16 and Plackett-Burman experiment design methods**

**Sahar Marghzari, ^1^ Mojtaba Sasani, ^2^ Massoud Kaykhaii, *^1, 3^ Mona Sargazi, ^1^ and Mohammad Hashemi ^4^**

^1^Department of Chemistry, Faculty of Sciences, University of Sistan and Baluchestan, Zahedan 98155-674, Iran

^2^Young Researchers and Elite Club, Zahedan Branch, Islamic Azad University, Zahedan, Iran

^3^Smartphone Analytical Sensors Research Centre, University of Sistan and Baluchestan, Zahedan, Iran

^4^Department of Clinical Biochemistry, School of Medicine, Zahedan University of Medical Science, Zahedan, Iran

**Table S1.** Factors and levels in Taguchi design to remove MG, RhB and CR

| Level | pH | Adsorbent dose (g) | NaCl added (g.mL^-1^) | Contact time (min) | Initial dye concentration (mg.L^-1^) |
| --- | --- | --- | --- | --- | --- |
| 1 | 6 | 0.50 | 0.025 | 10 | 3 |
| 2 | 7 | 1.25 | 0.050 | 20 | 6 |
| 3 | 8 | 2.00 | 0.075 | 30 | 9 |
| 4 | 9 | 2.50 | 0.100 | 40 | 12 |

**Table S2.** ANOVA results for simultaneous removal of MG, RhB and CR

| Parameter | DOF  (f) | | | Sum of square  (S) | | | Variance  (V) | | | Pure sum  (S’) | | | Percent  P (%) | | |
| --- | --- | --- | --- | --- | --- | --- | --- | --- | --- | --- | --- | --- | --- | --- | --- |
|  | MG | RhB | CR | MG | RhB | CR | MG | RhB | CR | MG | RhB | CR | MG | RhB | CR |
| pH | 3 | 3 | 3 | 1.407 | 0.204 | 1.22 | 0.469 | 0.068 | 0.406 | 1.407 | 0.204 | 1.22 | 39.352 | 3.09 | 5.787 |
| Adsorbent dosage (g) | 3 | 3 | 3 | 1.564 | 0.333 | 7.573 | 0.521 | 0.111 | 2.524 | 1.564 | 0.333 | 7.573 | 43.743 | 5.058 | 35.901 |
| NaCl added (g.mL^-1^) | 3 | 3 | 3 | 0.464 | 1.151 | 3.599 | 0.154 | 0.383 | 1.199 | 0.464 | 1.151 | 3.599 | 12.993 | 17.445 | 17.061 |
| Contact time (min) | 3 | 3 | 3 | 0.068 | 2.026 | 1.859 | 0.022 | 0.675 | 0.619 | 0.068 | 2.026 | 1.859 | 1.911 | 30.695 | 8.815 |
| Initial dye concentration (mg. L^-1^) | 3 | 3 | 3 | 0.071 | 2.885 | 6.841 | 0.023 | 0.961 | 2.28 | 0.071 | 2.885 | 6.841 | 1.996 | 43.969 | 32.428 |
| Other error | 0 | 0 | 0 | - | - | - | - | - | - | - | - | - | - | - | - |
| Total | 15 | 15 | 15 | 3.576 | 6.602 | 21.095 | - | - | - | - | - | - | 100 | 100 | 100 |

**Table S3.** Factors and levels were used for concurrent adsorption of MG, RhB and CR in PBD

| Parameter | -1 | +1 |
| --- | --- | --- |
| pH | 6 | 9 |
| Adsorbent dosage (g) | 0.5 | 2.5 |
| NaCl added (g.ml^-1^) | 0.025 | 0.100 |
| Contact time (min) | 10 | 40 |
| Initial dye concentration (mg. L^-1^) | 3 | 12 |

**Table S4.** Kinetic parameters of simultaneous removal of MG, RhB and CR by Sistan sand

| Kinetic model | Adsorbate | R^2^ | obtained parameters | |
| --- | --- | --- | --- | --- |
|  |  |  | K_1_ (min^-1^) | **q_e (cal)_ (mg g^-1^)** |
| Pseudo first order | MG | 0.9525 | 0.6757 | 0.4074 |
|  | CR | 0.9789 | 0.3408 | 0.2127 |
|  | RhB | 0.8565 | 0.2909 | 0.3560 |
| Pseudo second order | Adsorbate | R^2^ | K_2_ (g mg^-1^ min^-1^) | q_e (cal)_ (mg g^-1^) |
|  | MG | 0.9801 | 2.4740 | 0.1829 |
|  | CR | 0.9809 | 2.1518 | 0.1898 |
|  | RhB | 0.9948 | 1.0072 | 0.1975 |

**Table S5.** Thermodynamic parameters on simultaneous removal of MG, RhB and CR

|  | Temp ( ֯C) | ∆S֯ (J/mol K) | ∆H֯ (KJ/mol) | ∆G֯ (KJ/mol) |
| --- | --- | --- | --- | --- |
| MG | 25 | 74.99 | 25.555 | -27.430 |
|  | 30 |  |  | -27.805 |
|  | 40 |  |  | -28.555 |
|  | 50 |  |  | -29.305 |
| RhB | 25 | 47.08 | 18.436 | -19.613 |
|  | 30 |  |  | -19.848 |
|  | 40 |  |  | -20.319 |
|  | 50 |  |  | -20.790 |
| CR | 25 | 52.41 | 20.278 | -21.588 |
|  | 30 |  |  | -21.850 |
|  | 40 |  |  | -22.374 |
|  | 50 |  |  | -22.899 |


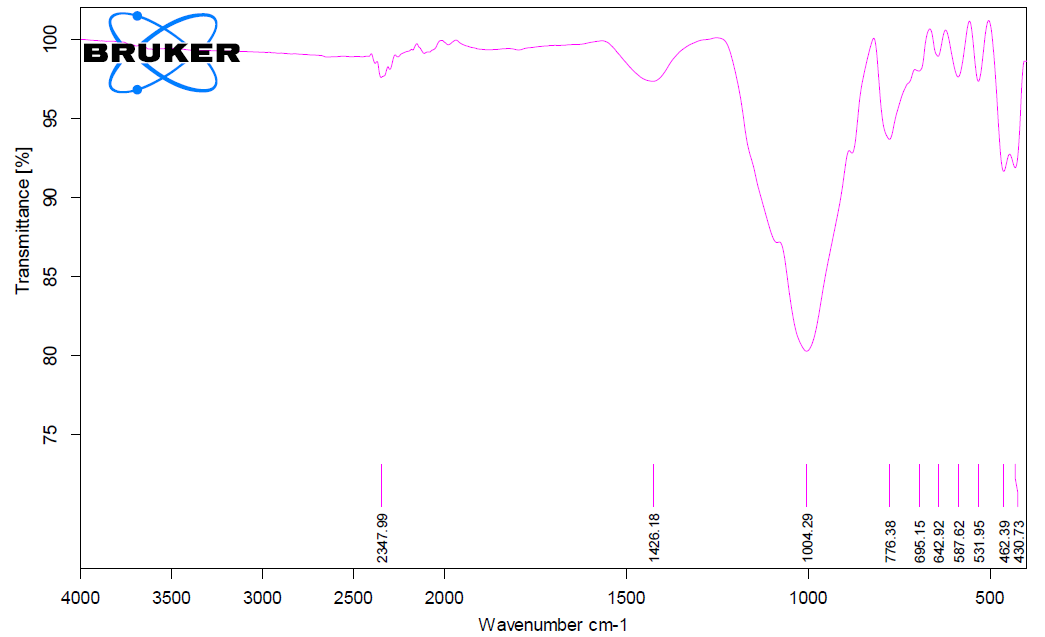
**Figure S1**. FT-IR of Sistan sand


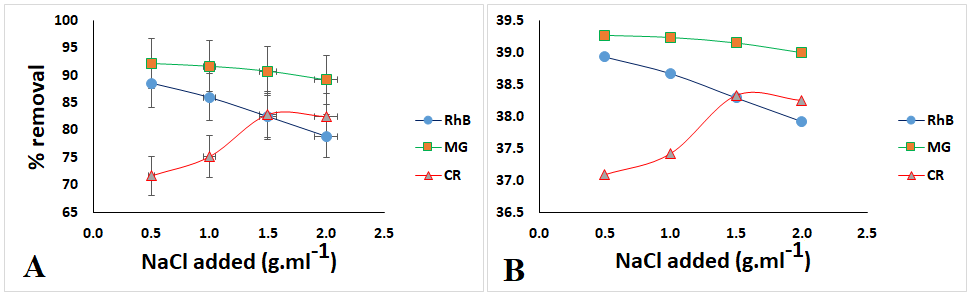


**Figure S2.** Effect of ionic strength on removal of MG, RhB and CR based on Mean (A) and S/N (B)


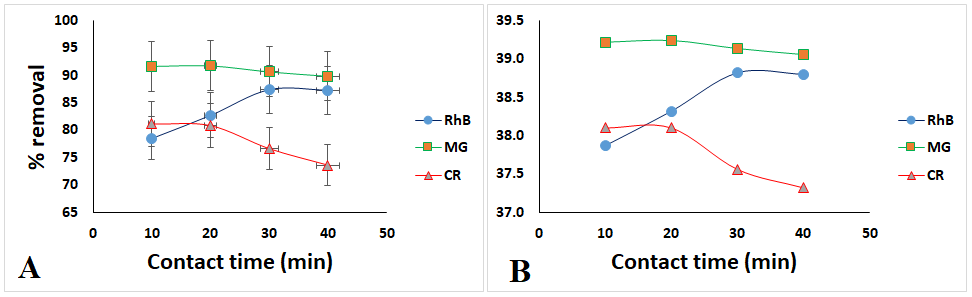


**Figure S3.** Effect of contact time on concurrent adsorption based on Mean (A) and S/N ratio (B)


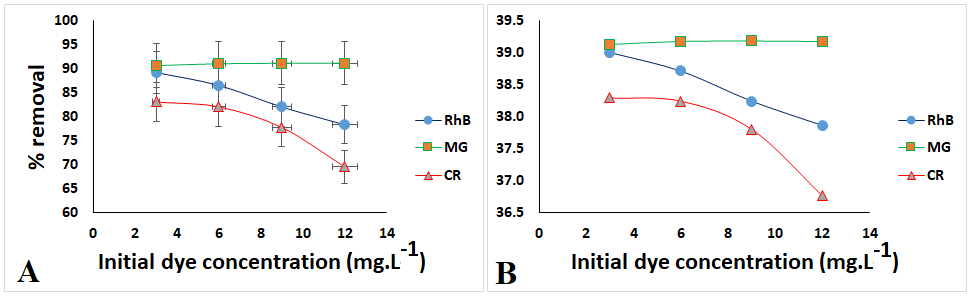


**Figure S4.** Effect of initial dye concentration on simultaneous adsorption based on Mean (A) and S/N ratio (B)


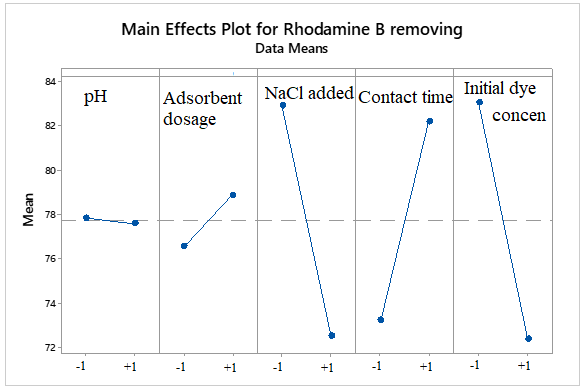


**Figure S5.** Main effects plot for RhB removal by PBD


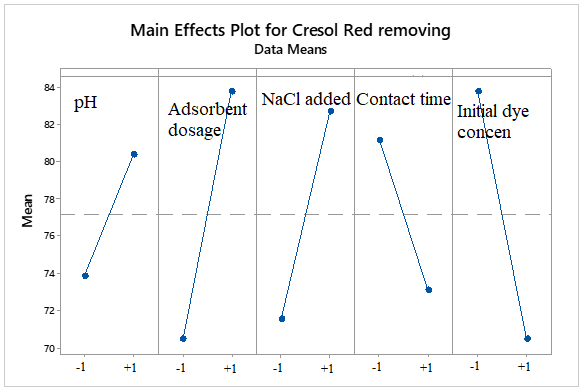


**Figure S6.** Main effects plot for CR removal by PBD
